# Connective tissue remodelling is differently modulated by tocilizumab versus methotrexate monotherapy in patients with early rheumatoid arthritis: the AMBITION study

**DOI:** 10.1186/s13075-020-02378-7

**Published:** 2021-01-07

**Authors:** Patryk J. Drobinski, Anne C. Bay-Jensen, Morten A. Karsdal, Samra Sardar, Anne S. Siebuhr

**Affiliations:** 1 ImmunoScience, Nordic Bioscience, Herlev Hovedgade 207, DK-2730 Herlev, Denmark; 2 Department of Biomedical Sciences, University of Copenhagen, Blegdamsvej 3, 2200 Copenhagen N, Denmark; 3 Biomarkers and Research, Nordic Bioscience, Herlev Hovedgade 207, DK-2730 Herlev, Denmark

**Keywords:** Tocilizumab (TCZ), Methotrexate (MTX), Rheumatoid arthritis (RA), Biomarkers, Tissue remodelling, Extracellular matrix (ECM)

## Abstract

**Objective:**

Associations between rheumatoid arthritis (RA) and effect of treatment at the tissue levels are poorly understood. We investigated the scope of released extracellular matrix (ECM) metabolites as a consequence of tissue remodelling in patients treated with methotrexate (MTX) and tocilizumab (TCZ) compared to placebo.

**Methods:**

Tissue metabolites from 387 RA patients treated with either TCZ (8 mg/kg) or MTX monotherapy (7.5–20 mg/kg) were measured at baseline and 8 weeks sera by validated ELISA assays. The levels of collagen biomarkers (C1M, C2M, C3M and C4M) together with C-reactive protein (CRP) and CRP metabolite (CRPM) were investigated. Baseline levels of biomarkers have been compared with 72 age- and gender-matched healthy controls. Comparison between treatment and response groups were done by ANCOVA, Spearman’s correlation and logistic regression adjusted for age, gender, BMI and disease duration.

**Results:**

C1M and C3M were significantly (*P* < 0.05) inhibited by TCZ and C3M by MTX (*P* < 0.01) compared to placebo. C1M and C3M inhibition with TCZ was respectively 23% and 16% greater than that of MTX (*P* < 0.01 and *P* < 0.0001). C4M was inhibited by TCZ and MTX, but the effect of TCZ was 22% greater than MTX (*P* < 0.0001). TCZ and MTX had minimal effect on C2M levels. MTX had no effect on CRP and CRPM, whereas TCZ reduced their levels to 69% and 27% from baseline. Investigated biomarkers revealed a significant (*P* < 0.05) difference in biomarker profiles of MTX ACR50 treatment responders and non-responders. Change to week 8 in levels of C3M, C4M, CRP and CRPM in MTX patients correlated significantly (rho = 0.41 to 0.18, *P* < 0.0001 to 0.039) with change in disease activity (DAS28) at weeks 8, 16 and 24, whereas only CRP in TCZ patients (rho = 0.32 to 0.21, *P* < 0.0001 to 0.01).

**Conclusion:**

Patients receiving TCZ treatment for 8 weeks had higher suppression of tissue remodelling and inflammatory biomarkers over patients treated with MTX. Measured biomarkers enabled for a discrimination of biomarker profiles of ACR50 treatment responding patients and identification of those who benefit at the early time point. Week 8 change in levels of C3M, C4M, CRP and CRPM significantly predicted clinical response to treatment and correlated with DAS28 at all time points.

**Trial registration:**

ClinicalTrials.gov, NCT00109408. Date of registration: July 2005. Name of the registry: A Study to Assess the Safety and Efficacy of Tocilizumab in Patients with Active Rheumatoid Arthritis.

## Introduction

Rheumatoid arthritis (RA) is a complex autoimmune, inflammatory disease, which primarily affects synovial joints and causes local damage along with systemic manifestations [1, 2]. It is prevalent in 0.5–1% of the world’s population. The disease has a progressive course leading to permanent disability and is therefore associated with extensive personal, social and economic impacts. Despite decades of research into this disease, the aetiology of RA is still not completely understood but is known to be dependent on both genetic susceptibility and environmental factors [3].

The introduction of disease-modifying anti-rheumatic drugs (DMARDs) such as methotrexate (MTX) has substantially changed the prognosis and improved everyday life of RA patients. MTX relieve disease symptoms and remains the most commonly used disease-modifying anti-rheumatic drug [4, 5]. However, therapies involving MTX often have inadequate response or intolerance, which calls for alternative treatments such as biological agents [6]. One of them is tocilizumab (TCZ), a humanised anti-IL-6 receptor monoclonal antibody, which acts by blocking IL-6 signalling [7–9]. Elevated levels of IL-6 in synovial fluid and serum correlate with disease activity in patients with RA [10, 11]. Inhibition of the IL-6 signalling by TCZ showed a protective effect on bone erosions by decreasing their volume in RA patients and significant reduction of swollen and tender joint counts with concurrent improvement in radiographic progression [9, 12].

Key histological features of RA affected joints include hyperplasia and cellular infiltration of the synovium and systemic inflammation [13]. The inflammation leads to accelerated remodelling of extracellular matrix (ECM) that can progress to bone erosion and cartilage destruction [14, 15]. Destruction of connective tissue, especially the ECM of cartilage, bone and soft tissues of the joint, is a hallmark of RA pathology [16].

Proteolytic enzymes expressed locally in the pathologically affected tissue, such as the matrix metalloproteinases (MMPs) and aggrecanases, have a role in the remodelling of the ECM.

In healthy connective tissues, physiologic expression of these enzymes is low, but increases considerably during the inflammation process in RA thereby leading to excessive ECM remodelling [17]. We have previously shown that MMP-3 was highly elevated in RA and treatment with TCZ significantly decreased MMP3 levels [18]. The consequence of MMPs upregulation is heightened ECM remodelling with the release of a range of protein-specific degradation products (neo-epitopes) to circulation that can be used as biomarkers of tissue remodelling in joint diseases [19]. Former findings have shown that upregulated levels of MMP-degraded type I, II, III and IV collagen (C1M, C2M, C3M and C4M, respectively) correlated with disease activity in RA patients and treatment with TCZ+MTX significantly suppressed the biomarkers dose-dependently [20–23]. C1M was shown not only to be associated with disease activity, but a useful tool for identification of RA patients with fast structural progression [22]. Gudmann et al. have shown that C4M together with C1M and C3M are biomarkers reflecting structural joint degradation in RA and their baseline elevated levels significantly correlates with disease activity score of 28 joints (DAS28) [23].

Another type of biomarkers is biomarkers that indicate an ongoing inflammatory process. C-reactive protein (CRP), a biomarker of systemic inflammation, is produced and released from the liver as an acute reactant during inflammation, whereas CRPM, an MMP-degraded fragment of CRP, is released from the inflamed tissue. CRP levels have shown predict clinical outcomes from treatment with TCZ in RA and many other inflammatory diseases [24]. It was reported that levels of CRPM correlate with RA activity at different disease stages and significantly downregulate together with collagen biomarkers after TCZ+MTX treatment [21, 25, 26]. Serological biomarkers of tissue remodelling and inflammation may give insight into treatment effect at the tissue level of different interventions giving an alternative approach to currently used methods such as symptomatic changes assessed by ACR-response or DAS28. In our study, we investigated if tissue remodelling was differently modulated by TCZ and MTX monotherapies in RA patients by measuring tissue remodelling biomarkers.

## Materials and methods

### Patients and study design

**Fig. 1:**
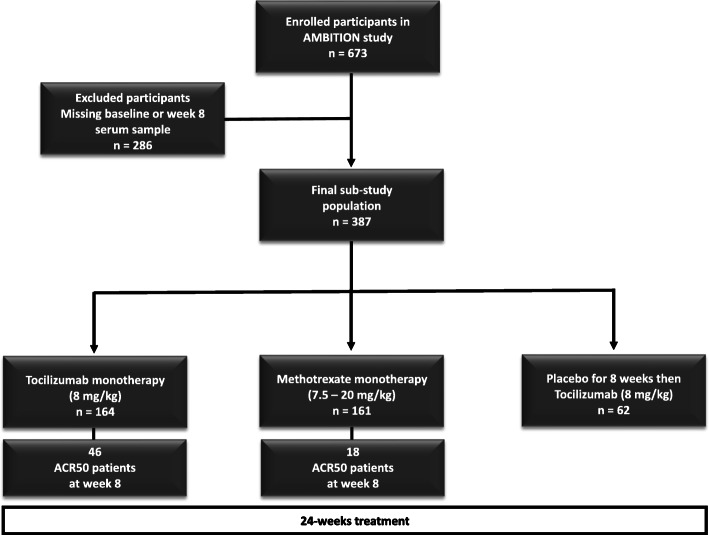
Patient randomisation into three different treatment arms in the AMBITION study

The AMBITION study (NCT00109408) was a 24-week phase 3 trial, double-blind, double-dummy, parallel-group, including 673 biological naive patients, randomised to either tocilizumab (TCZ) monotherapy 8 mg/kg intravenously every 4 weeks or methotrexate (MTX) monotherapy oral capsules every week (initial dose 7.5 mg and titrated to 15 mg at week 4 and to 20 mg at week 8) [27]. Placebo (PBO) patients received tocilizumab 8 mg/kg from week 8 until the end of the study. The current biomarker sub-study of AMBITION involved baseline and week 8 serum samples from a total number of 387 adult patients (> 18 years) with moderate to severe active RA (Fig. 1). The AMBITION study was approved by the ethics committee at each participating institution (in USA, Canada and Israel) and was conducted in accordance with the principles of good clinical practice and according to the Declaration of Helsinki. All patients included provided written, informed consent before inclusion in the study. Serum from 72 healthy controls included in the study was obtained from the Discovery Life Sciences, Inc., vendor with compliance to the Ethics Committee recommendations and all regulations, guidelines and best practices that meet or exceed the US and international regulatory requirements.

### Biochemical marker assays

Serum levels of type I [28], II [29], III [30] and IV [31] collagen degradation biomarkers together with CRP and its metabolite CRPM [25] were measured in serum by technically validated ELISAs developed by Nordic Bioscience (Herlev, Denmark). Biomarkers were measured according to manufacture instructions. A brief description of the protocol follows: Streptavidin-coated 96-well plates were coated with respective biotinylated antigens for 30 min at 20 °C and subsequently washed 5 times in washing buffer. Appropriate controls, standard and serum samples were added with subsequent addition of peroxidase conjugated antibody against the respective epitope.

The mixture was incubated depending of an assay for 1 h at 20C (C3M, C4M, CRPM) or 20 h (reC1M, C2M) at 4 °C. Plates were subsequently washed 5 times with washing buffer. TMB substrate (3,3′,5,5′-tetramethylbenzidine) was added and incubated for 15 min at 20 °C and the reaction was stopped with 0.18 M sulphuric acid. A SpectraMax Microplate Reader (Molecular Devices Corporation, Sunnyvale, CA, USA) was used to read the absorbance at 450 nm with reference set to 650 nm. The concentrations were calculated by using a 4-parametric curve fit model. Biomarker results were approved under fulfilling acceptance requirements of an analytical run. This comprised the acceptance of three main criteria. Firstly, acceptance of the standard curve with all standards points resulting in < 10% coefficient of variance (CV) and < 15% of relative error (%RE) within the analytical measuring range. The exception from that criteria was that one standard point could be masked if CV > 10% or if %RE > 15%. Secondly, acceptance of the quality controls with all resulting in < 15% CV for all QC specimens and within the target range of mean ± 20%, with the exception that one QC specimen was allowed to have CV > 15%. Lastly, acceptance of sample measurement for samples within the measurement range and < 15% CV. Samples with a CV > 15% were re-analysed.

### Statistical analyses

**Fig. 2:**
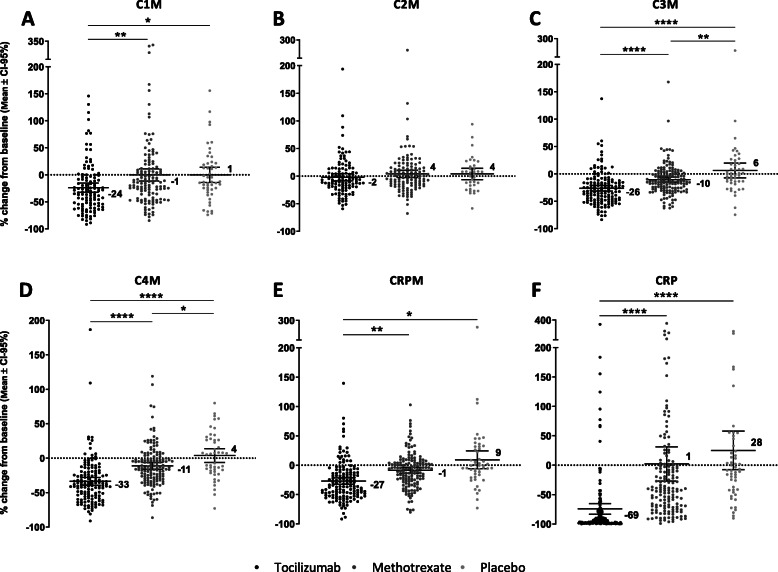
Figures represent differences in modulation of tissue remodelling biomarkers C1M (**a**) (TCZ *n* = 115; MTX *n* = 128; PBO *n* = 49), C2M (**b**) (TCZ *n* = 109; MTX *n* = 122; PBO *n* = 36), C3M (**c**) (TCZ *n* = 141; MTX *n* = 145; PBO *n* = 47), C4M (**d**) (TCZ *n* = 140; MTX *n* = 146; PBO *n* = 46), CRPM (**e**) (TCZ *n* = 143; MTX *n* = 145; PBO *n* = 48) and CRP (**f**) (TCZ *n* = 164; MTX *n* = 161; PBO *n* = 53) according to treatment from baseline to week 8 (%). Data are shown as mean with CI-95%

The distribution of patient’s baseline parameters among different treatment groups was assessed by one-way ANOVA *t* test and presented in Table 1. Biomarker values that were above (ULOD) detection range were remeasured in dilution or excluded from the further analysis. The lower detection limit (LLOD) values were handled by setting the detection limit cutoff for confident determination of sample concentration and statistical differentiation from a blank at a 99% confidence level. Change in biomarker levels at week 8 in relation to baseline was tested by paired *t* test for each treatment (Fig. 2). Comparison between treatment groups (TCZ, MTX and PBO) was examined by ANCOVA at baseline and week 8, adjusted for age, gender, BMI and disease duration.

**Table 1:** Patient demographics and baseline characteristics of the AMBITION cohort, according to treatment group. Differences between treatment groups are presented by one-way ANOVA *t* test

Characteristics	Tocilizumab, ***N*** = 164	Methotrexate, ***N*** = 161	Placebo, ***N*** = 62	***P*** value
**Age (years), mean (SD)**	50.7 (12.9)	51.3 (13.3)	50.5 (11.8)	ns
**Female,** ***n*** **(%)**	131 (80)	122 (76)	47 (76)	ns
**Male,** ***n*** **(%)**	33 (20)	39 (24)	15 (24)	ns
**DAS28, mean (SD)**	6.8 (0.9)	6.8 (0.9)	6.9 (0.9)	ns
**BMI (SD)**	28.1 (6.4)	27.4 (6.0)	29.1 (7.4)	ns
**TJC, mean (SD)**	32.6 (14.8)	32.1 (14.1)	35.4 (16.3)	ns
**SJC, mean (SD)**	19.4 (10.9)	19.9 (10.6)	23.5 (12.2)	*P* = 0.04
**RADUR (SD)**	6.7 (8.1)	6.6 (8.0)	7.4 (9.0)	ns
**CRP (mg/dL), mean (SD)**	3.2 (3.6)	3.1 (3.5)	2.5 (2.7)	ns
**ESR (mm/h), mean (SD)**	46.9 (26.1)	47.9 (24.1)	48.2 (22.4)	ns
**HAQ-DI, mean (SD)**	1.6 (0.6)	1.6 (0.6)	1.4 (0.5)	ns
**Pain VAS 100 mm, mean (SD)**	59.0 (22.4)	61.3 (20.7)	59.5 (24.4)	ns
**Patient VAS 100 mm, mean (SD)**	64.6 (22.0)	64.9 (19.4)	64.3 (22.5)	ns
**Physician VAS 100 mm, mean (SD)**	63.5 (16.1)	64.6 (16.3)	69.1 (17.9)	ns
**Baseline C1M (ng/mL), mean (95% CI)**	42.7 (37.5–47.8)	45.2 (39.4–50.9)	40.5 (34.5–46.5)	ns
**Baseline C2M (ng/mL), mean (95% CI)**	0.4 (0.4–0.5)	0.4 (0.4–0.5)	0.3 (0.3–0.4)	ns
**Baseline C3M (ng/mL), mean (95% CI)**	17.8 (16.5–19.2)	18.3 (17.1–19.5)	15.9 (13.8–17.9)	ns
**Baseline C4M (ng/mL), mean (95% CI)**	53.9 (49.3–58.4)	51 (47.2–54.8)	42.3 (36.5–48.1)	*P* = 0.02
**Baseline CRPM (ng/mL), mean (95% CI)**	21.9 (19.6–24.2)	20.8 (18.9–22.8)	18.5 (15.0–21.9)	ns
**Baseline CRP (ng/mL), mean (95% CI)**	3.2 (2.6–3.8)	3.1 (2.6–3.7)	2.5 (1.8–3.2)	ns

*DAS28* 28-Joint Disease Activity Score, *TJC* tender joint count, *SJC* swollen joint count, *CRP* C-reactive protein, *ESR* erythrocyte sedimentation rate, *HAQ-DI* Health Assessment Questionnaire-Disability Index, *VAS* visual analogue scale

Correlation between the change in biomarker levels from baseline to week 8 and disease activity (DAS28) at weeks 8, 16 and 24 was assessed with Spearman’s correlation (rho) (Table 4). The normality of data distribution was assessed by Shapiro-Wilk test and significance between groups was assessed by Mann-Whitney nonparametric test. Data were presented as a mean with either 95% confidence interval (CI-95%), standard error of mean (SEM) or standard deviation of mean (SD). Statistical significance in mean biomarker change between treatment groups at baseline and week 8 were assessed by unpaired ANOVA *t* test. Significance was considered when *P* values were *P < 0.05, **P < 0.01, ***P < 0.001, ****P < 0.0001. All statistical analyses were performed by using MedCalc version 14.8.1 and Prism Graphpad version 8.1.2 for graphical preparation.

## Results

### Baseline demographics

This current sub-study of AMBITION had 387 patients divided into three treatment arms with similar distribution of age, BMI, gender and disease activity (Table 1). There were no significant differences in baseline parameters between treatment groups except the number of swollen joint count (SJC) that was slightly higher in placebo patients (*P* = 0.04) and lower baseline C4M levels in placebo patients (*P* = 0.02). The same differences regarding clinical parameters were found in the complete AMBITION study (data not shown). All biomarkers except C2M correlated significantly at baseline (*P* < 0.0001 to *P* < 0.01, Spearman’s rho 0.15 to 0.31) to the initial DAS28. The baseline levels of biomarkers in sub-study of AMBITION cohort were compared with healthy controls, involving 72 age- and gender-matched donors (Table 2). Comparison of these values was performed with ANCOVA adjusted for race differences and revealed significantly lower baseline levels of all biomarkers in healthy controls (*P* < 0.001).

**Table 2:** Comparison of AMBITION cohort with healthy controls in patient demographics and baseline biomarker levels. Differences between cohorts are shown as ANCOVA values adjusted for race differences

Variables	AMBITION cohort RA patients (***N*** = 387)	Healthy controls (***N*** = 72)	Difference between cohorts
**Age (years), mean (SD)**	50.9 (12.9)	49.7 (6.1)	ns
**Female,** ***n*** **(%)**	300 (76)	41 (76)	ns
**Race, no. white/black/other (%)**	325 (84)/14 (4)/48 (12)	51 (71)/21 (29)/0 (0)	< 0.0001
**Baseline C1M (ng/mL), mean (SD)**	43.4 (30.7)	18.7 (15.3)	< 0.001
**Baseline C2M (ng/mL), mean (SD)**	0.38 (0.2)	0.3 (0.16)	< 0.001
**Baseline C3M (ng/mL), mean (SD)**	17.8 (8.0)	11.1 (2.8)	< 0.001
**Baseline C4M (ng/mL), mean (SD)**	50.9 (24.9)	21.5 (6.1)	< 0.001
**Baseline CRPM (ng/mL), mean (SD)**	20.9 (12.9)	7.5 (2.1)	< 0.001
**Baseline CRP (ng/mL), mean (SD)**	3.0 (3.5)	–

**Table 3:** Treatment-dependent difference in core outcome measures used to assess disease activity in rheumatoid arthritis (RA)

Parameter, % change from baseline to week 8	ACR50 Patients			
	Tocilizumab		Methotrexate	
	Responders	Non-Responders	Responders	Non-Responders
DAS28% mean (CI-95%)	− 54.7 (− 60.1 to −49.3)	−30.0 (− 33.0 to − 27.0)	−46.4 (− 52.1 to − 40.7)	−12.8 (− 15.7 to − 10.1)
SJC, % mean (CI-95%)	− 78.4 (− 83.8 to − 73.0)	−29.7 (− 37.6 to − 21.7)	−80.7 (− 89.0 to − 72.5)	−22.8 (− 32.1 to − 13.6)
TJC, % mean (CI-95%)	−76.3 (− 80.7 to − 72.0)	−25.8 (− 34.1 to − 17.5)	−73.6 (− 81.4 to − 65.8)	− 22.1 (− 30.1 to − 14.2)
VAS Pain, % mean (CI-95%)	−7.7 (− 35.2 to 19.9)	−10.5 (− 28.5 to 7.5)	−40.8 (− 68.3 to − 13.4)	−22.8 (− 36.6 to − 8.9)

Table represent mean percentage change from baseline to week 8 for each respective parameter. *DAS28* 28-Joint Disease Activity Score, *TJC* tender joint count, *SJC* swollen joint count, *VAS Pain* visual analogue scale pain

### Biomarker modulation at week 8

The week 8 levels of biomarkers associated with connective tissue remodelling, C1M and C3M were significantly downregulated by tocilizumab (TCZ) when compared to placebo (*P* < 0.05 and *P* < 0.0001, respectively) and C3M also by methotrexate (MTX) compared to placebo (*P* < 0.01) (Fig. 2a, c). The inhibition of C1M and C3M with TCZ was, respectively, 23% and 16% greater than that of MTX (*P* < 0.01 and *P* < 0001). The levels of the basement membrane remodelling biomarker C4M were likewise inhibited by both TCZ and MTX comparing to placebo (*P* < 0.0001 and *P* < 0.05, respectively) and the effect of TCZ was 22% greater than MTX (*P* < 0.0001) (Fig. 2d). In contrast, TCZ had minimal and MTX no effect on downregulation of the cartilage degradation biomarker C2M when compared to placebo (Fig. 2b). Considering effect of MTX on inflammatory biomarkers modulation, both CRP and CRPM levels were downregulated at week 8 when compared to placebo, 27% and 10% decrease, respectively (Fig. 2e, f). TCZ showed greater than MTX inhibitory effect on CRP and CRPM resulting in 97% (*P* < 0.0001) and 36% (*P* < 0.01) decrease comparing to placebo patients.

### Treatment-dependent difference in biomarker profiles of ACR50 responders and non-responders

Patients of the TCZ and MTX groups who achieved ACR50 at week 8 were classified as treatment responders. Patients that did not meet ACR50 at week 8 were classified as treatment non-responders. The treatment with TCZ demonstrated to be better than MTX with a higher ACR50 response rate (*P* = 0.01) (Table 3). Among the 164 patients that were treated with TCZ, 46 patients reached ACR50 at week 8 (28%). In the MTX group, only 18 of the 161 patients reached ACR50 at week 8 (11%).

**Fig. 3:**
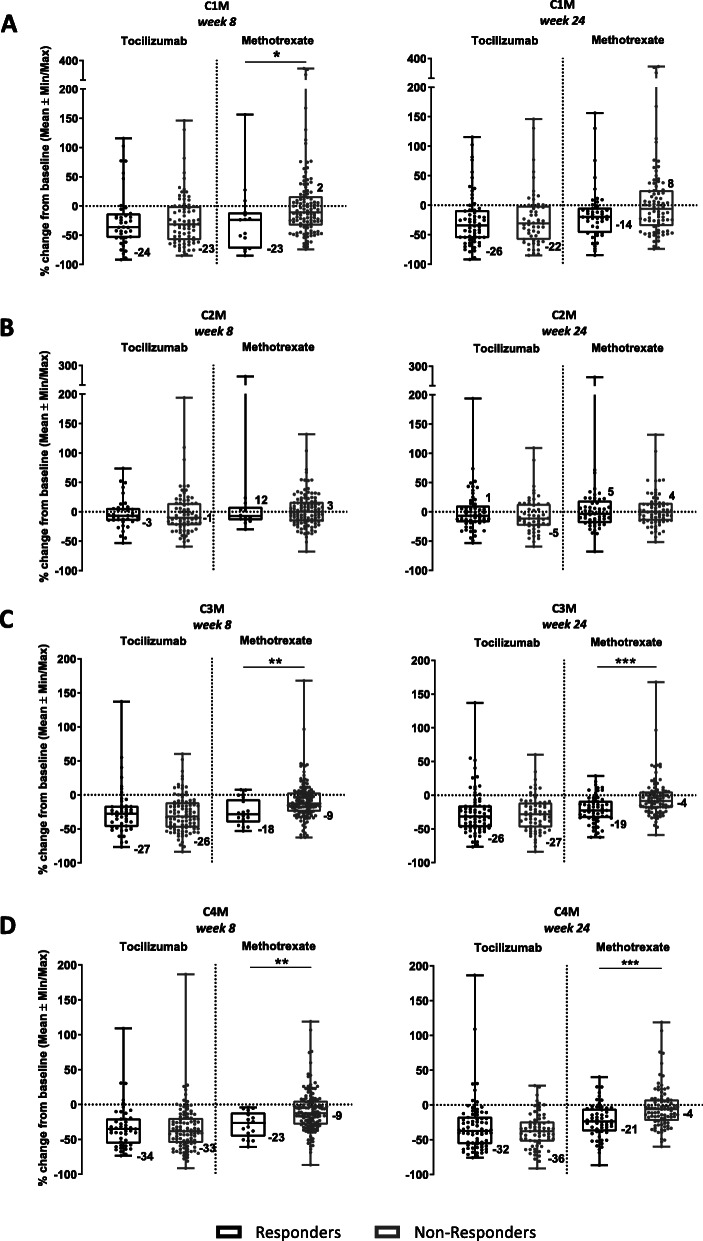
Treatment-dependent difference in degradation biomarker profiles of patients assigned as ACR50 responders (black bars) and ACR50 non-responders (grey bars) from baseline to week 8 and predictive profiles at week 24. Figures represent serum levels of C1M (**a**) (TCZ *n* = 115; MTX *n* = 128), C2M (**b**) (TCZ *n* = 109; MTX *n* = 122), C3M (**c**) (TCZ *n* = 141; MTX *n* = 145) and C4M (**d**) (TCZ *n* = 140; MTX *n* = 146) in response to TCZ and MTX at week 8 and predictive profiles at week 24. Data are shown as mean with SEMs

C1M among responders and non-responders in TCZ group did not show significant difference in the biomarker profiles at weeks 8 and 24, whereas MTX patients showed significant (*P* < 0.05) difference in C1M levels at week 8 (23% decrease vs 2% increase) (Fig. 3a). There was a clear difference in profiles of C1M MTX patients once correlated to DAS28 at week 24, albeit statistically insignificant. Treatment with either TCZ or MTX did not result in significant differences in C2M profiles of responding and non-responding patients at weeks 8 and 24 (not significant (ns)) (Fig. 3b). MTX showed a trend to increase the C2M level, whereas the level was unchanged with TCZ. C3M profiles did not display any significant differences after treatment with TCZ between responders and non-responders at week 8 and 24; however, MTX patients showed significant difference (*P* < 0.01) in the decrease of C3M levels at week 8 (18% in responders vs 9% in non-responders) (Fig. 3c). The statistical significance between MTX C3M profiles increased (*P* < 0.001) once correlated with DAS28 at week 24 (19% decrease in responders vs 4% in non-responders). Similarly, treatment with TCZ did not result in significantly different C4M profiles in responders and non-responders at both time points, while MTX patients displayed significant difference (*P* < 0.01) in the decrease of C4M levels at week 8 (23% in responders and 9% in non-responders) with increased significance (*P* < 0.001) once correlated with DAS28 at week 24 (21% in responders and 4% in non-responders) (Fig. 3d).

**Fig. 4:**
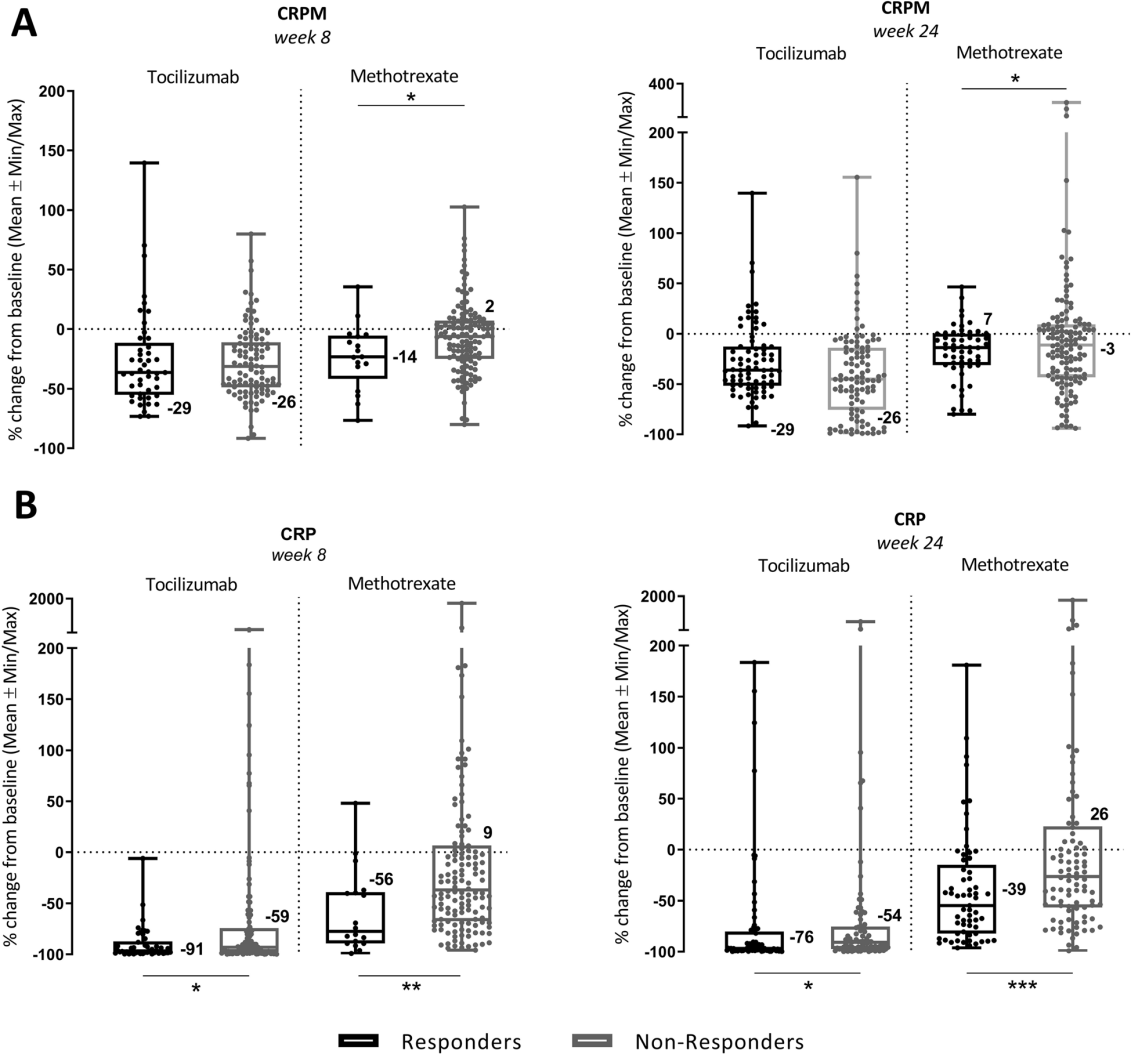
Treatment-dependent difference in inflammatory biomarker profiles of patients assigned as ACR50 responders (black bars) and ACR50 non-responders (grey bars) from baseline to week 8 and predictive profiles at week 24. Figures represent serum levels of CRPM (**a**) (TCZ *n* = 143; MTX *n* = 145) and CRP (**b**) (TCZ *n* = 164; MTX *n* = 161) in response to TCZ and MTX at week 8 and predictive profiles at week 24. Data are shown as mean with SEMs

Treatment with TCZ did not result in significantly different CRPM profiles among responders and non-responders at weeks 8 and 24, but MTX patients displayed significantly different (*P* < 0.05) CRPM profiles at week 8 (14% decrease in responders vs 2% increase in non-responders) (Fig. 4a). Intriguingly, the opposite profiles of CRPM was observed once correlated with DAS28 at week 24, 7% increase in responders and 3% decrease in non-responders in CRPM levels (*P* < 0.05).

There was observed a strong modulation of the CRP levels among both treatment groups. Patients treated with TCZ displayed a significantly different (*P* < 0.05) decrease of CRP profiles between responders and non-responders at week 8 (91% vs 59%) and after correlation to DAS28 at week 24 (76% vs 54%) (Fig. 4b). Similarly, treatment with MTX resulted in significantly different (*P* < 0.01) CRP profiles between responders and non-responders at week 8 (56% decrease vs 9% increase) with increased significance (*P* < 0.001) after correlation to DAS28 at week 24 (39% decrease vs 26% increase; Table 3).

### Correlation between change in biomarker levels and change in disease activity at weeks 8, 16 and 24

To investigate whether differences in tissue degradation and tissue specific inflammation levels can be associated with overall improvement in disease activity, the difference in biomarker levels were correlated to the change in disease activity score (DAS28) at weeks 8, 16 and 24. The prognostic value of measured biomarkers was assessed by Spearman’s rank correlation coefficient (rho) and *P* value between change in tissue remodelling biomarkers from baseline to week 8 and change in disease activity (DAS28) at week 16 and 24 (Table 4). Correlation values account for Bonferroni correction in order to avoid type I error rate per biomarker. Significance has been adjusted for number of tests performed for each respective biomarker. In this case, significance of *P* < 0.05 was adjusted to *P* < 0.0083. As a validation step, biomarker levels at baseline in the TCZ group were correlated to DAS28 change at week 16. These findings were validated by investigating correlation between week 8 biomarker levels with DAS28 change at week 24 in the PBO group. None of the investigated biomarker correlated to DAS28 in either TCZ or PBO group and no significant differences in correlation between both groups were observed (data not shown). In patients treated with tocilizumab (TCZ), only CRP was significantly correlated with change in DAS28 at week 8 (rho = 0.32, *P* < 0.0001) and week 16 (rho = 0.31, *P* = 0.0001) (Table 4). In patients treated with methotrexate (MTX), three biomarkers correlated with change in DAS28 at all time points (C3M, C4M, CRP). Change in C3M levels correlated to change in DAS28 at week 8 with correlation coefficient of 0.32 (*P* = 0.0001) at week 8, 0.3 (*P* = 0.0005) at week 16 and 0.26 (*P* = 0.003) at week 24. Change in levels of C4M correlated with change in DAS28 with correlation coefficient of 0.35 (*P* < 0.0001) at week 8, 0.32 (*P* = 0.0001) at week 16 and 0.26 (*P* = 0.002) at week 24. Change in levels of CRP correlated with change in DAS28 with correlation coefficient of 0.41 (*P* < 0.0001) at week 8, 0.34 (*P* < 0.0001) at week 16 and 0.25 (*P* = 0.0026) at week 24. Moreover, change in levels of CRPM correlated significantly with change in DAS28 with correlation coefficient of 0.29 (*P* = 0.0006) at week 8.

**Table 4:** Correlation between week 8 change in levels of respective biomarkers according to the treatment group and change in disease activity (ΔDAS28) at weeks 8, 16 and 24. Correlation values are given as Spearman’s correlation coefficient (rho) and significance as *P* value. Significant correlations with *P* < 0.0083 have been highlighted (Bonferroni correction)

Biomarker	Tocilizumab			Methotrexate		
	ΔDAS28 week 8	ΔDAS28 week 16	ΔDAS28 week 24	ΔDAS28 week 8	ΔDAS28 week 16	ΔDAS28week 24
**C1M**	rho = 0.08	rho = 0.06	rho = 0.002	rho = 0.11	rho = 0.06	rho = 0.08
	*P* = 0.43	*P* = 0.53	*P* = 0.98	*P* = 0.22	*P* = 0.53	*P* = 0.4
**C2M**	rho = 0.02	rho = 0.03	rho = 0.02	rho = 0.04	rho = − 0.04	rho = 0.02
	*P* = 0.87	*P* = 0.73	*P* = 0.83	*P* = 0.66	*P* = 0.69	*P* = 0.87
**C3M**	rho = 0.1	rho = 0.12	rho = 0.05	**rho = 0.32**	**rho = 0.3**	**rho = 0.26**
	*P* = 0.24	*P* = 0.16	*P* = 0.54	***P*** **= 0.0001**	***P*** **= 0.0005**	***P*** **= 0.003**
**C4M**	rho = 0.08	rho = 0.08	rho = − 0.01	**rho = 0.35**	**rho = 0.32**	**rho = 0.26**
	*P* = 0.38	*P* = 0.37	*P* = 0.89	***P*** **< 0.0001**	***P*** **= 0.0001**	***P*** **= 0.002**
**CRP**	**rho = 0.32**	**rho = 0.31**	rho = 0.21	**rho = 0.41**	**rho = 0.34**	**rho = 0.25**
	***P*** **< 0.0001**	***P*** **= 0.0001**	*P* = 0.01	***P*** **< 0.0001**	***P*** **< 0.0001**	***P*** **= 0.0026**
**CRPM**	rho = 0.13	rho = 0.13	rho = 0.1	**rho = 0.29**	rho = 0.18	rho = 0.21
	*P* = 0.13	*P* = 0.14	*P* = 0.24	***P*** **= 0.0006**	*P* = 0.039	*P* = 0.016

## Discussion

In this sub-study of the AMBITION trial, we investigated association of different tissue remodelling biomarkers with aspects of joint degradation and inflammation in RA. We investigated the biomarker profiles in response to TCZ, MTX and placebo. In the full AMBITION study, clinical signs and symptoms after treatment with TCZ and MTX have been examined, but tissue remodelling biomarkers have not been investigated [27]. The approach involving tissue remodelling biomarker assessment gives a unique insight into the drug’s mode of action at the tissue level and ongoing tissue remodelling processes.

Results of this study confirmed that investigated biomarkers are significantly upregulated in rheumatoid arthritis (Table 2), suggesting that an ongoing excessive ECM remodelling results in the excessive release of these biomarkers from the affected joint structures to circulation.

We demonstrated that there was significant difference between the two treatments and placebo patients in modulation of tissue remodelling biomarkers. Levels of C1M, C3M and C4M were significantly suppressed by TCZ compared to MTX, suggesting that TCZ exhibit more tissue protective effect towards different joint structures than that of MTX. TCZ had a minimal and MTX no effect on cartilage degradation measured by C2M. We observed a great inhibition of CRP and significant inhibition of CRPM after treatment with TCZ, whereas MTX had limited effect on their levels. This can be explained by the fact that IL-6 receptor is a main target for TCZ treatment and CRP is produced in the liver in the response to high levels of IL-6. Thus, TCZ should suppress CRP, which is observed and further downstream suppression of CRPM, which is observed as expected. The superior effect of TCZ in controlling of CRP and CRPM levels in the present study can be compared with LITHE study where TCZ inhibited completely the level of CRP and in the large extent level of CRPM [20]. These results indicate that TCZ is more effective than MTX treatment that better limits tissue turnover and inflammation. More importantly, it indicates that measured biomarkers can be successfully applied to reveal differences in the mode of action between the investigated treatments.

We examined biomarker profiles of ACR50 responding and non-responding patients to investigate if any of the biomarker could stratify patients that benefited from treatments already at week 8. The significant differences in tissue remodelling biomarker profiles between treatment responders and non-responders were observed primarily among patients treated with MTX. This was an interesting observation considering that MTX patients had less biomarker downmodulation than TCZ patients. Despite the fact that TCZ patients were more likely to reach ACR50 than MTX patients, the significant differences in biomarker profiles discriminating responders from non-responders were observable only for CRP. Previously, similar studies have shown that combination treatment involving TCZ+MTX do not necessarily display differences in profiles of tissue turnover biomarkers between ACR50 responders and non-responders already at week 8 [20]. These differences were observable at weeks 16 and 24, which could explain results seen in TCZ patients. Interestingly, results of this study showed that the separation of these treatments allowed to observe differences in biomarker profiles of patients treated with MTX monotherapy as early as at week 8. This indicates that the neo-epitope biomarkers can be potentially used as a tool to identify treatment responding and non-responding patients at the early treatment time point. As a possible explanation behind the observed differences in biomarker profiles might be different mechanisms underlying the mode of action of TCZ and MTX. TCZ limits the inflammation by selective inhibition of IL-6 receptors and forming the receptor-complex that prevents IL-6 signal transduction that stimulates B and T cells. MTX as opposed to TCZ is a broad spectrum, systemic immune system suppressant acting on a wide range of immune cells and cytokines production. As a consequence of these treatment-dependent differences, joint structures might be potentially differently modulated and result in distinct biomarker profiles between treatments.

Biomarkers of tissue turnover have shown a great prognostic potential and can predict long-term clinical response to treatment already at the early time point [26]. It is of great importance to determine at the possibly earliest stage whether patient will respond to the treatment in the future and benefit from the treatment. We demonstrated that only change to week 8 in the CRP levels significantly predicted clinical response to TCZ and correlated with the change in the disease activity at weeks 16 and 24 as a response to treatment (Table 4). Interestingly, more biomarkers were predictive for MTX treatment. In this case, change to week 8 in the C3M, C4M, CRP and CRPM levels significantly predicted clinical response at weeks 16 and 24.

These results show a great value of the biomarkers which can be a determining indicator of patient clinical benefit from the given treatment. Previous studies have shown that biomarker levels reflecting structural joint damage such as C1M, C2M and C3M can be associated with disease progression and disease activity over time [22, 32].

Our results confirm similar biomarker analysis performed for LITHE study that measured C2M, C3M, CRPM and CRP, but in patients treated with combination therapy involving TCZ at two doses (8 and 4 mg/kg) with MTX. The combination therapy of TCZ and MTX decreased C3M and CRPM levels at week 8 in a comparable extent to TCZ alone in our study [20]. There was a greater decrease in levels of C2M and CRP at week 8 after TCZ+MTX treatment, albeit TCZ monotherapy significantly decreased levels of CRP proving its positive effect on inflammation process. We observed a limited effect of TCZ alone on cartilage balance measured with C2M; however, high C2M upregulation is typical at erosive disease stages and that was not a case in the examined cohort. Another study involving the LITHE cohort investigated the effect of structural joint damage and progression, measured with C1M [22]. TCZ in combination with MTX decreased levels of C1M at week 8 in a greater extent than TCZ monotherapy, but both treatments provided significant downmodulation of C1M and protective effect on connective tissues. Gudmann et al. investigated the baseline membrane remodelling measured with C4M after TCZ+MTX treatment in LITHE and RADIATE cohorts [23]. Reported results support our findings, where TCZ+MTX treatment downregulated levels of C4M at week 8 in the similar extent as TCZ monotherapy, proving that both treatment strategies have protective effect on tissue remodelling. These results indicate that TCZ monotherapy can be successfully applied as an effective alternative to commonly used combined therapy involving TCZ+MTX. One of the main limitations of this study was that we did not measure biomarkers at other time points than baseline and week 8, so we could not anticipate the long-term treatment effect on biomarker modulation. Another limitation of this study was no radiographic to support our findings of tissue remodelling. However, radiographic showed sensitivity limitations in detecting early structural changes in joints and surrounding structures, indicating their limited applicability in this type of study [33].

## Conclusion

Patients receiving TCZ treatment for 8 weeks have clearly higher suppression of tissue remodelling and inflammatory biomarkers over patients treated with MTX. The decrease of the inflammatory process is believed to be associated with decrease of ECM remodelling and tissue degradation, which is supported by this study. On the other hand, patients receiving MTX enable for a clear discrimination of biomarker profiles and identification of those who were most likely to benefit from the treatment at the early time point. The results of this study highlight that biomarkers reflecting tissue turnover can be applied as disease and response to therapy markers. Their application may facilitate future identification of patients most likely to benefit from a given treatment with the aim to develop possibly best personalised treatment strategy.

## Data Availability

The datasets used and/or analysed during the current study are available from the corresponding author on reasonable request.

## Abbreviations

ACR50: 50% disease improvement
BMI: Body mass index
C1M: Type I collagen degradation biomarker
C2M: Type II collagen degradation biomarker
C3M: Type III collagen degradation biomarker
C4M: Type IV collagen degradation biomarker
CRP: C-reactive protein
CRPM: C-reactive protein degradation biomarker
CV: Coefficient of variance
DAS28: Disease Activity Score in 28 joints
DMARDs: Disease-modifying anti-rheumatic drugs
ECM: Extracellular matrix
IL-6: Interleukin-6
LLOD: Lower limit of detection
MMP: Matrix metalloproteinase
MTX: Methotrexate
PBO: Placebo
RA: Rheumatoid arthritis
TCZ: Tocilizumab
TMB: 3,3′,5,5′-Tetramethylbenzidine substrate
ULOD: Upper limit of detection
